# 810-nm Photobiomodulation Evokes Glutamate Release in Normal and Rotenone-Dysfunctional Cortical Nerve Terminals by Modulating Mitochondrial Energy Metabolism

**DOI:** 10.3390/cells14020067

**Published:** 2025-01-07

**Authors:** Silvia Ravera, Elisa Farsetti, Guido Maura, Manuela Marcoli, Matteo Bozzo, Chiara Cervetto, Andrea Amaroli

**Affiliations:** 1 Department of Experimental Medicine, University of Genova, 16132 Genova, Italy; silvia.ravera@unige.it; 2 IRCCS Ospedale Policlinico San Martino, 16132 Genova, Italy; 3 Department of Pharmacy, Section of Pharmacology and Toxicology, University of Genova, 16148 Genova, Italy; elisa.farsetti@edu.unige.it; 4 Department of Earth, Environment and Life Sciences (DISTAV), University of Genova, 16132 Genova, Italy; guido.maura@gmail.com (G.M.); manulea.marcoli.maura@gmail.com (M.M.); matteo.bozzo@unige.it (M.B.); 5 Interuniversity Center for the Promotion of the 3Rs Principles in Teaching and Research (Centro 3R), 56122 Pisa, Italy; 6 BIO-Photonics Overarching Research Laboratory, Department of Earth, Environmental and Life Sciences (DISTAV), University of Genova, 16132 Genova, Italy

**Keywords:** synaptosomes, glutamate, low-level light therapy, low-level laser therapy, phototherapy, oxidative phosphorylation, mitochondrial metabolism, mitochondrial dysfunction, neuron degenerations, neurotoxic disorder

## Abstract

The dysfunction of mitochondria, the primary source of cellular energy and producer of reactive oxygen species (ROS), is associated with brain aging and neurodegenerative diseases. Scientific evidence indicates that light in the visible and near-infrared spectrum can modulate mitochondrial activity, a phenomenon known in medicine as photobiomodulation therapy (PBM-t). The beneficial effects of PBM-t on dementia and neurodegeneration have been reviewed in the literature. However, the molecular mechanisms underlying these findings have yet to be fully elucidated. This study investigates the mechanism behind dose-dependent glutamate release in nerve terminals after irradiation with 810 nm, 1 W for 60 s continuous, 1 cm^2^, 1 W/cm^2^, 60 J, 60 J/cm^2^ (810 nm-1 W) or 810 nm, 0.1 W for 60 s continuous, 1 cm^2^, 0.1 W/cm^2^, 6 J, 6 J/cm^2^ (810 nm-0.1 W), focusing on mitochondrial activities. The results show that PBM modulated the mitochondrial metabolism of cortical nerve terminals and supported a power-dependent increase in oxidative phosphorylation (OxPhos) activity when stimulated with pyruvate plus malate (P/M) or succinate (succ) as respiratory substrates. The PBM-induced increase in OxPhos was sensitive to adding rotenone (Complex I inhibitor) and antimycin A (Complex III inhibitor) when synaptosomes were stimulated with P/M, but only to antimycin A when stimulated with succ. This allowed us to observe that the glutamate efflux, disrupted in the presence of rotenone, was partially restored by PBM due to the increase in the OxPhos pathway led by Complex II. This evidence suggests that PBM, acting on mitochondria, could facilitate physiological communication within the neuron-astrocyte network through vesicular glutamate release, potentially regulating healthy brain function and brain dysfunction.

## 1. Introduction

Photobiomodulation therapy (PBM-t) is based on the ability to manipulate the metabolism of cells using non-ionizing electromagnetic radiation and non-thermal energies in the visible and near-infrared light range, up to about 1000 nm [1]. This treatment method exploits specific molecules’ ability in the animal cell to absorb energy from photons and then transfer it to support chemical reactions [2]. One of the key players in PBM is the mitochondrion [3]. The studies of Karu [4,5,6], followed by those of Passarella et al. [7,8], using a wavelength of around 630 nm, triggered the growing interest in stimulating mitochondria by visible light irradiation over the last 40 years. More recent studies [9,10] have further explored the possibility of activating photoacceptors in mitochondrial complexes using a range of wavelengths in the near-infrared spectrum. The photo-energization of cytochromes was shown to increase ATP production. In particular, diode laser light at 810 nm [11] or 980 nm [12] was observed to mainly affect Complex IV of the mitochondrial respiratory chain, with a minor effect on Complex III, while laser light at 1064 nm [13] stimulated Complexes I, III, and IV. This ability to convert photonic (physical) energy into chemical (ATP) energy has been employed in both human and veterinary medicine to support recovery from various diseases commonly linked to mitochondrial dysfunction [14,15,16,17,18,19].

Indeed, alterations in mitochondrial activity, often associated with respiratory chain dysfunction, have been implicated in pathogenesis [20]. This has been mainly observed in tissues with high energy demands, such as the central nervous system (CNS). Deficits in mitochondrial function are associated with cognitive decline and dementia [21]. Impaired mitochondrial function, which is the primary source of cellular energy and produces reactive oxygen species (ROS), has been linked to brain aging and the onset of diseases such as Alzheimer’s disease (AD) [22] and sporadic Parkinson’s disease (PD) [23]. It is important to note that even in the latent period before the onset of symptoms and diagnosis of AD, known as mild cognitive impairment, a significant mitochondrial imbalance can be detected compared to healthy subjects. However, there is no significant increase in senile plaques and neurofibrillary tangles [24]. In addition, studies on AD and PD have shown abnormalities in the mitochondrial electron transport chain complexes, leading to reduced energy efficiency. These changes may contribute to a negative feedback loop, also supported by increased ROS production, leading to further mitochondrial damage and an irreversible neurodegenerative cascade [25,26,27,28,29]. There is increasing evidence that glutamate plays an active role in the etiology of AD and PD, in addition to alterations in mitochondrial activity [30,31]. Glutamate is an important transmitter in CNS, the primary excitatory neurotransmitter in mammalians with significant roles in learning and memory [32], in the neurodevelopment and related disorders [33] as in the pathogenesis of neurodegenerative diseases, including AD and PD [34], and excitotoxic damage through an over-activation of its receptors. Nonetheless, glutamatergic transmission is commonly controlled by a strict relationship between neurons and astrocytes [35,36]. The glutamate transmission is highly dependent on calcium homeostasis and mitochondrial function [37,38,39,40,41,42].

AD and PD have distinct pathological and clinical features. However, they share specific mechanisms that contribute to neuronal damage. Higher cognitive processes involving the cortex and hippocampus are affected by alterations in the glutamatergic system at the presynaptic and postsynaptic levels and in astrocytes. This disruption is directly associated with cognitive impairment in AD [39,43,44]. Several lines of evidence support the idea of a pathological accumulation of Aβ-dependent glutamate in the diseased brain. This leads to neuronal hyperactivity. From this point of view, in the early stages of Alzheimer’s disease, when Aβ-dependent glutamate accumulation is observed, anti-glutamatergic treatment strategies could be beneficial in modifying the course of the disease [45]. Alternate glutamatergic transmission and dysfunctional neuronal metabolism are also central to the pathophysiology of PD. *Substantia nigra*’s susceptibility to glutamate neurotoxicity suggests a role for glutamate in the pathogenesis of PD. This susceptibility is exacerbated by a possible impairment of the mitochondria [37,46].

The interesting clinical results obtained with transcranial PBM could be explained by these close links between mitochondrial and glutamatergic dysfunction on the one hand and PBM and tissue photo-energizing mitochondria on the other. Indeed, the therapy has been shown to enhance cognition in both sick and healthy subjects [15,47,48,49,50] and patients with emotional learning disorder and major depression [16].

However, the molecular mechanisms underlying these findings have yet to be fully elucidated.

In a previous study, we showed that PBM irradiated with an 810-nm diode laser set to deliver 1 W of laser light continuously for 60 s over a 1 cm^2^ area (1 W/cm^2^, 60 J, 60 J/cm^2^) evoked a release of glutamate from cortical nerve terminals at levels with neurotoxic potential [51]. However, a lower power of 0.1 W induced glutamate release in a physiological range from nerve terminals. This was similar to that measured by near-physiological stimulation [52]. Overall, PBM appeared to have dose-dependent effects related to calcium flux and membrane depolarization [52].

In this work, we investigated the mechanism underlying dose-dependent glutamate release in nerve terminals after irradiation with 810-nm, 1 W for 60 s continuous, 1 cm^2^, 1 W/cm^2^, 60 J, 60 J/cm^2^ (hereafter referred to as 810 nm-1 W) or with 810-nm, 0. 1 W for 60 s continuously, 1 cm^2^, 0.1 W/cm^2^, 6 J, 6 J/cm^2^ (hereafter referred to as 810 nm-0.1W for brevity), with a focus on mitochondrial activities. The study shows how PBM acts on the mitochondria of cortical nerve terminals. It supports a power-dependent increase in ATP production. The inhibition by rotenone and antimycin is consistent with the hypothesis that mitochondrion and ATP production are critical points in the effect of PBM on endogenous glutamate. Furthermore, glutamate efflux evoked by near-physiological stimulation was deregulated entirely in the presence of rotenone. However, the neurotransmitter release was partially restored by stimulation with PBM. The evidence supports that PBM treatment, acting on mitochondria, could facilitate vesicular glutamate release in the neuron-astrocyte network. This could also be a regulator of healthy brain function and brain dysfunction.

## 2. Materials and Methods

### 2.1. Materials

Percoll^®^, 4-aminopyridine (4-AP), rotenone, and *o*-phthalaldehyde were from Sigma-Aldrich (Milan, Italy). All the salts used for the standard HEPES medium were from VWR (Milan, Italy). 4-AP and rotenone were dissolved in distilled water and then diluted in HEPES medium.

### 2.2. Animals

Male C57BL/6J mice aged 3 months were used. They were housed 3–4 in a cage with free access to standard food and water ad libitum under constant environmental conditions (22 ± 1 °C; 50% humidity; lights on 7 a.m.–7 p.m.). Animal care and handling were by Italian law (D.L. 26/2014) and the European Directive (2010/63/EU). They were approved by the Italian Ministry of Health (protocol n° 75F11.N.0RF, of November 2021) and by the Unit for the Welfare of Laboratory Animals of the University of Genova, Genova, Italy. Every effort was made to minimize the number of mice used in this experimental research.

### 2.3. Preparation of Synaptosomes

Synaptosomes are resealed presynaptic terminals prepared as previously described [52]. Cortices were homogenized in 0.32 mM sucrose solution buffered with Tris at pH 7.4. The homogenate was centrifuged, and the supernatant was stratified on a discontinuous Percoll^®^ gradient (2, 6, 10, 20 % (*v/v*) in Tris-buffered sucrose) and centrifuged. Synaptosomes were collected at the 10-20% (*v/v*) Percoll layers and were suspended in standard HEPES medium (mM: NaCl 128, KCl 2.4, MgSO_4_ 1.2, KH_2_PO_4_ 1.2, CaCl_2_ 1.0, HEPES 10, glucose 10, pH 7.4). As previously described, synaptosomes are a purified preparation of nerve terminals assessed by immunofluorescence using antibodies to specific markers for astrocytes, microglia, and oligodendrocytes [53]. In addition, synaptosomes are equipped with the machinery for the vesicular release of neurotransmitters and often contain mitochondria in the cytoplasm to sustain the high demand for ATP required for exocytosis and maintain ionic gradients [52].

### 2.4. Technical Characteristics of the Equipment Used and the Irradiation Setup

Synaptosomes were irradiated during superfusion or in bulk (glass cuvette).

Irradiations were performed with an ENEA GaAl-As diode laser (Garda Laser, 7024 Negrar, Verona, Italy). The device was calibrated to allow irradiation at a wavelength of 810-nm ± 2 for a specific duration of 60 s in continuous wave mode.

The irradiation power was adjusted to 1.0 or 0.1 W for treated samples, ensuring a consistent energy output of 60 or 6 J (power density of 1.0 or 0.1 W/cm^2^; fluence of 60 or 6 J/cm^2^).

For control samples, the power was set to 0 W. Irradiations were delivered using a flat-top profiled handpiece (FT-HP), which was previously characterized [54]. The FT-HP provides uniform energy distribution over a 1 cm^2^ spot area independent of distance [54,55]. In both treatments, a 635-nm red light pointer with a negligible power output of less than 0.5 mW visualized the exposed area, ensuring precise targeting of the irradiation.

A Pronto-250 power meter (Gentec Electro-Optics, Inc. G2E Quebec City, QC, Canada) was used to ensure the accuracy of the irradiated laser parameters.

**Figure 1:**
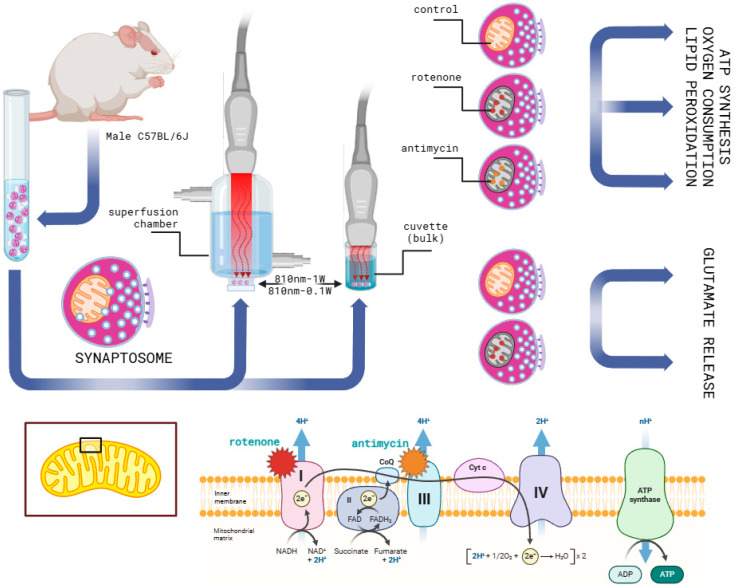
Brief experimental setup description. A mouse model was used to obtain cortical nerve terminals (synaptosomes). After being collected in test tubes, they were irradiated with a diode laser at 810 nm with the parameters described in Section 2.4. The synaptosomes, key to our energy metabolism and oxidative stress experiments, were irradiated at the bottom of a glass cuvette. The synaptosomes were carefully placed on a filter at the bottom of a superfusion chamber for the glutamate release experiments. A power meter was meticulously used to ensure uniform irradiation under both experimental conditions. To assess the role of mitochondrial metabolism in PBM-evoked glutamate release, samples were treated with rotenone and antimycin, inhibitors of mitochondrial respiratory chain Complexes I and III, respectively. The effect of PBM on dysfunctional synaptosomes was tested using rotenone treatment.

The irradiation was carried out with the handpiece fixed in contact mode with a cuvette or the perfusion chamber (Figure 1). As previously described [56], the cuvette is cylindrical with a flat bottom. The thickness of the glass, the total height, and the external diameter of the chambers were 1.2 mm, 1.2 cm, and 1.5 cm, respectively. The bottom of the perfusion chamber, where the synaptosomes are placed on a filter, is similar in size to the cuvette.

Monitoring the exposure with a FLIR ONE Pro-iOS thermal camera (FLIR Systems, Inc., Portland, OR, USA) (dynamic range: −20 °C/+400 °C; resolution 0.1 °C) avoided unwanted thermal effects.

### 2.5. Evaluation of OxPhos Activity and Efficiency

The oxygen consumption rate (OCR) and ATP synthesis through FoF1 ATP-synthase were evaluated as markers of oxidative phosphorylation (OxPhos) activity using 50 μg of total protein from mouse cortex synaptosomes, lasered or not, permeabilized with 0.03 mg/mL digitonin immediately before use.

Oxygen consumption was assessed with an amperometric oxygen electrode within a closed, magnetically stirred chamber at 25 °C. The samples were resuspended in PBS, and 5 mM pyruvate plus 2.5 mM malate (P/M) or 20 mM succinate (succ) to stimulate the pathway involving Complexes I, III, and IV, or Complexes II, III, and IV, respectively. In each case, 0.1 mM ADP was added [57].

Aerobic ATP production was determined using samples dissolved in a solution containing 10 mM Tris-HCl (pH 7.4), 50 mM KCl, 1 mM EGTA, 2 mM EDTA, 5 mM KH_2_PO_4_, 2 mM MgCl_2_, 0.6 mM ouabain, and 0.040 mg/mL ampicillin. The reaction was monitored using the luciferin/luciferase method (luciferin/luciferase ATP bioluminescence assay kit CLSII, Roche, Basel, Switzerland) at 25 °C following the addition of the same respiratory substrates employed for OCR measurements. The reaction started with the addition of 0.1 mM ADP and was monitored by the GloMax^®^ 20/20 Luminometer (Promega, Madison, WI, USA) for over two minutes, with measurements taken every 30 s [57].

For both assays, when necessary, 10 μM rotenone or 10 μM antimycin A (ant-A) was employed to inhibit Complex I or Complex III, respectively.

The ratio between the aerobically synthesized ATP and the consumed oxygen (P/O ratio) was calculated to evaluate OxPhos efficiency. A value around 2.5 (for P/M stimulation) or 1.5 (for succ stimulation) indicates complete coupling between energy production and respiration [58], while lower values suggest an uncoupling state that favors reactive oxygen species (ROS) formation [59].

### 2.6. Evaluation of Lipid Peroxidation

Lipid peroxidation levels were determined as an oxidative damage marker by evaluating malondialdehyde (MDA) using the thiobarbituric acid reactive substances (TBARS) assay. The TBARS solution comprised 15% trichloroacetic acid in 0.25 N HCl and 26 mM 2-thiobarbituric acid. To determine the basal MDA concentration, 600 μL of TBARS solution was added to 50 μg of total protein from mouse cortex synaptosomes, lasered or not, dissolved in 300 μL of water. The samples were incubated at 95 °C for 40 min, then centrifuged at 20,000× *g* for 2 min, and the supernatant was analyzed spectrophotometrically at 532 nm [57].

### 2.7. Superfusion Experiments and Endogenous Glutamate Assessment

To study the glutamate release, we performed superfusion at 37 °C on equal aliquots of synaptosomes transferred in parallel superfusion chambers. The up-down superfusion with standard HEPES medium of a synaptosomal monolayer at a flow rate of 0.5 mL/min guarantees the complete and continuous removal of the molecules released in the biophase of the presynaptic targets and a fast and easy exchange of the medium [51,52]. This experimental approach allows the study of the direct effects of light irradiation on glutamate release from cortical nerve terminals [51,52]. After 33 min of perfusion, necessary to re-equilibrate the basal efflux, four superfusate fractions were collected every 3 min. At t = 38 min, synaptosomes were exposed to light irradiation with the second experimental setup (810 nm–0.1W) or to 4-aminopyridine (4-AP 300 µM, 3 min) in the absence or presence of rotenone 10 µM starting 6 min before irradiation or depolarising stimulation. At t = 41 min, the medium was changed and maintained until the end of the experiment. In each experiment, at least one chamber was not exposed to light or 4-AP and used as a control, and at least one chamber was exposed to rotenone alone and used as a rotenone basal control. When 4-AP and laser were applied together during superfusion, irradiation was applied immediately after 4-AP stimulation; after 1 min, when the laser light was switched off, 4-AP stimulation continued for a further 2 min. At the end of the chemical stimulation, the physiological medium was rapidly replaced with a fresh buffer for further superfusion until the end of the experiment.

Reverse HPLC measured the glutamate present in the collected fractions of superfused synaptosomes using an automated precolumn derivatization with a solution of o-phthalaldehyde, separation on a C18 column, and a gradient mobile phase. The amount of glutamate in each fraction was then calculated as pmol/mg protein. For each chamber, the sum of the first two fractions was considered the basal release, and the irradiation/chemical stimulation-evoked glutamate efflux (overflow) was measured by subtracting the basal release from the total amount of glutamate released during and after stimulation.

### 2.8. Statistical Analysis

The data in the graphs are expressed as means ± SEM of the n experiments as indicated throughout. Data were analyzed using one-way ANOVA followed by Tukey’s multiple comparisons test or *t*-test. The significance of differences at *p* < 0.05 was taken to indicate statistical significance.

## 3. Results

### 3.1. PBM Increases OxPhos Activity Without Affecting Its Efficiency

**Figure 2:**
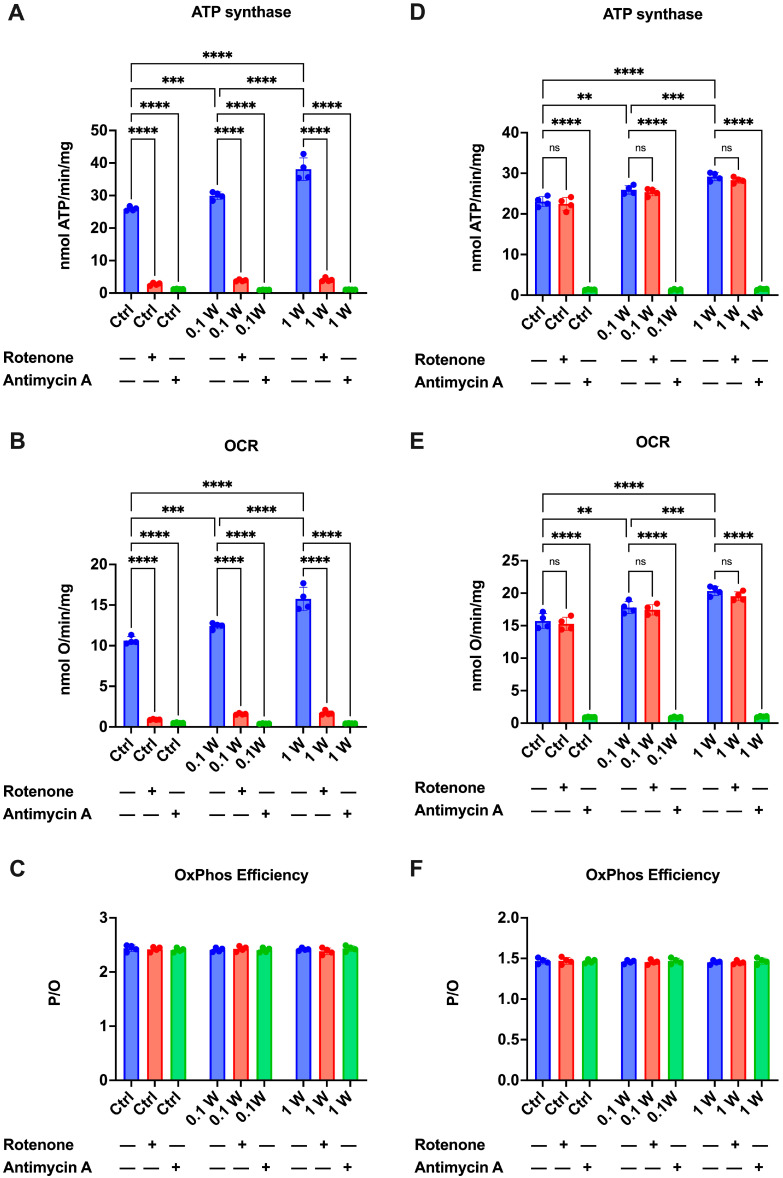
PBM effect on OxPhos activity of cortical synaptosomes. (**A**) Pyruvate plus malate (P/M)-induced ATP synthesis; (**B**) P/M-induced Oxygen Consumption Rate (OCR); (**C**) P/M-induced P/O value as an OxPhos efficiency marker; (**D**) Succinate (Succ)-induced ATP synthesis; (**E**) Succ-induced Oxygen Consumption Rate (OCR); (**F**) Succ-induced P/O value. All experiments were conducted in the absence (blue bars) or presence of 10 μM rotenone (red bars) or 10 μM antimycin A (green bars) to inhibit Complex I and Complex III, respectively. Data are represented as mean ± SEM and are presentative of four independent experiments. Significant differences were tested by one-way ANOVA followed by Tukey’s multiple comparisons test. **, ***, and **** indicate a *p* < 0.01, *p* < 0.001 or *p* < 0.0001, ns indicates a non-statistically significant difference, respectively.

Aerobic energy synthesis and respiration were measured to evaluate the impact of PBM on the OxPhos metabolism of synaptosomes. The data show that laser treatment caused an increase in both ATP synthesis (Figure 2A,D) and OCR (Figure 2B,E) compared to the untreated sample, in a dose-dependent manner, as the increase was significantly higher in the sample treated with 1 W (about 46% vs. ctrl) compared to the one that received 0.1 W (about 15% vs. ctrl). Furthermore, it is noteworthy that the increase in OxPhos functionality due to laser treatment did not affect the coupling between energy production and cellular respiration, as the P/O ratio, a marker of mitochondrial energy production efficiency, remained consistent with the reference value [58] (Figure 2C, i.e., around 2.5 in the presence of P/M and 1.5 with succinate).

Although the presence of aerobic metabolism in synaptosomes has been previously reported [57,60], to confirm that the observed ATP synthesis and OCR were due to OxPhos, the experiment was also conducted in the presence of rotenone and antimycin A, specific Complex I and Complex III inhibitors, respectively. As shown in Figure 1, adding these inhibitors in pyruvate/malate (P/M)-induced OxPhos virtually reduced both ATP synthesis and OCR to zero in both the control and PBM-treated samples, confirming that synaptosomal energy production and respiration depend on the OxPhos activity. In succinate-stimulated metabolism, only antimycin A abolished energy production (Figure 2D) and respiration (Figure 2E), as rotenone does not inhibit Complex II activity.

### 3.2. OxPhos Activity Increment Was Not Associated with Oxidative Damage Accumulation

**Figure 3:**
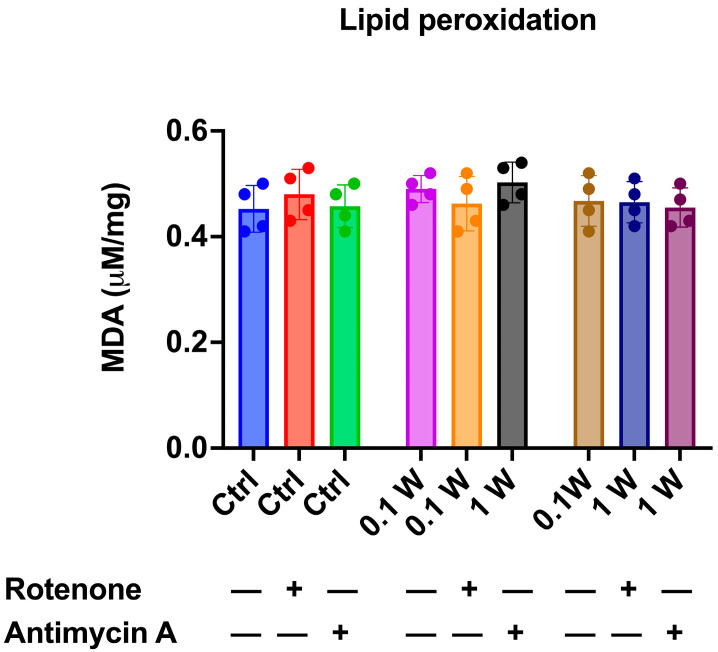
Lipid peroxidation accumulation in cortical synaptosomes after PBM. The graph shows the intracellular MDA concentration as a lipid peroxidation marker. The experiments were conducted in the absence (blue bars) or presence of 10 μM rotenone (red bars) or 10 μM antimycin A (green bars) to inhibit Complex I and Complex III, respectively. Data are represented as mean ± SEM and are representative of four independent experiments. Statistical analysis was performed by one-way ANOVA followed by Tukey’s multiple comparisons test, and no significant differences were observed.

Given that aerobic metabolism is a significant source of oxidative stress even when ATP synthesis and respiration are perfectly coupled [61], we assessed the impact of PBM-induced OxPhos enhancement on oxidative damage accumulation by measuring intracellular MDA concentration as a marker of lipid peroxidation. The data presented in Figure 3 show that despite the increase in OxPhos following laser treatment (Figure 1), the laser-treated synaptosomes did not exhibit a higher accumulation of MDA than the untreated ones, confirming the coupling between ATP synthesis and OCR. Notably, adding rotenone and antimycin A did not alter the MDA concentration, even though the inhibition of OxPhos would be expected to decrease the production of reactive oxygen species. This apparent discrepancy can be explained by the fact that rotenone and antimycin A were added just before the laser treatment and lipid peroxidation assessment. Therefore, the MDA levels accumulated at the synaptic level are still detectable in the inhibited samples.

### 3.3. PBM Induced Endogenous Glutamate Release in Cortical Nerve Terminals and Controlled the Depolarization-Evoked Excessive Efflux in Rotenone-Dependent Mitochondrial Dysfunction

#### 3.3.1. PBM-Induced Endogenous Glutamate Release in Cortical Nerve Terminals in the Presence of Rotenone

Previously, we observed that laser irradiation at 1 W and 0.1 W induced glutamate release from cortical synaptosomes in a power-dependent manner, and the effect on the glutamate release evoked by 0.1 W was similar to that observed using a quasi-physiological stimulation [52]. Here, we investigated the PBM effects in the presence of a significant reduction in ATP production due to Complex I inhibition by rotenone.

**Figure 4:**
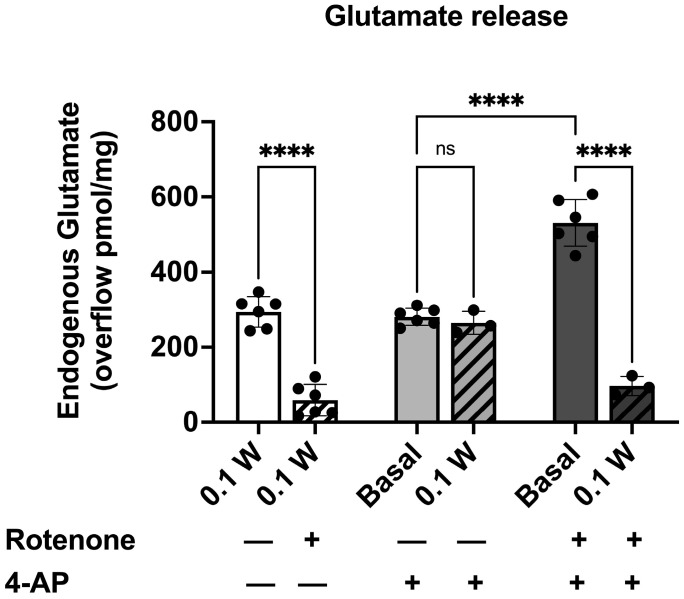
Release of glutamate from cortical synaptosomes in the absence or the presence of rotenone: effects of photons in basal and physiological stimulation. Effects of photons (0.1 W, 60 s) on the basal release of glutamate in superfused cortical synaptosomes in the absence (white bar) or in the presence of rotenone (10 µM, white stripped bar). 4-AP (300 µM, 3 min–grey bar) -evoked glutamate overflow and effect of the co-application of photons (0.1W, 60 s–stripped grey bar). Effect of rotenone on the 4-AP (300 µM, 3 min–dark grey bar) -evoked glutamate overflow and counteracting impact of photons (0.1 W, 60 s–stripped dark grey bar). Bars represent the overflow of glutamate release, expressed as pmol/mg of protein, in the different experimental conditions reported in the legend. Photons were applied for 60 s in basal condition or during the 4-AP stimulation; rotenone was added 6 min before the stimulation and maintained till the end of the experiment. Further experimental details can be found in Materials and Methods. Data are expressed as mean ± SEM of n = 3 to 6 independent experiments. **** *p* < 0.001 by one-way ANOVA followed by multiple comparisons test. ns indicates a non-statistically significant difference.

In our experimental conditions, the basal samples collected from cortical isolated nerve endings during superfusion with physiological standard medium had a glutamate efflux equivalent to 82.23 ± 4.20 pmol/mg protein min (n = 10). Stimulation with laser 0.1 W for 60 s during superfusion increased the efflux and evoked glutamate overflow (Figure 4). The presence of rotenone during superfusion did not affect basal release but produced a significant reduction in the endogenous glutamate efflux evoked by laser irradiation (Figure 4).

#### 3.3.2. PBM Did Not Affect 4-AP-Induced Endogenous Glutamate Release

During superfusion, the PBM effect was assessed when synaptosomes were exposed to a quasi-physiological stimulation, 4-AP (300 µM). 4-AP is known as a potassium channel blocker, and it may better mimic physiological excitation than an elevated KCl level. The mechanism of action of 4-AP seems linked to Na^+^-channel firing or Ca^2+^ entry [62,63,64]. 4-AP evoked a glutamate overflow significantly higher than the basal release (280.84 ± 9.38 pmol/mg protein; **** *p* < 0.001 compared to the basal release according to *t*-test; n = 6; Figure 4). The co-stimulation of cortical nerve terminals with 4-AP and photons induced a release of the neurotransmitter significantly different from the basal efflux (264.86 pmol/mg protein; **** *p* < 0.001 compared to the basal release according to *t*-test; n = 3; Figure 4) but not different from the laser-evoked or the 4-AP-evoked glutamate release (Figure 4).

#### 3.3.3. PBM Affected the 4-AP-Induced Endogenous Glutamate Release in Cortical Nerve Terminals Exposed to Rotenone

To investigate the potential therapeutic application of PBM in the neurological condition characterized by mitochondrial dysfunction, we repeated the experiments co-applicating both the laser and the depolarizing stimulations in the presence of rotenone. The effect of the reduction in ATP production due to rotenone application during superfusion was assessed when synaptosomes were exposed to 4-AP. The pre-treatment of synaptosomes with rotenone added 6 min before the depolarizing stimulus significantly modified the 4-AP-evoked neurotransmitter efflux (Figure 4). The collected data indicated that when the experiment was conducted in the presence of rotenone, a specific inhibitor of Complex I, the 4-AP-evoked release of glutamate became deregulated with a significant increase in overflow.

The effect of the PBM was then investigated on depolarized nerve terminals in the rotenone-induced reduction of ATP synthesis. The pre-treatment of synaptosomes with rotenone increased the 4-AP-evoked neurotransmitter efflux (Figure 4), but the co-application of the laser (0.1W, 60 s) significantly reduced the glutamate release (Figure 4).

## 4. Discussion

PBM at 810 nm modulates neurotransmission in synaptosomes via energy metabolism.

The beneficial effects of PBM-t on dementia and neurodegeneration have been extensively reviewed in the literature [65,66]. Our study further supports these findings by demonstrating that PBM can modulate the release of glutamate, a neurotransmitter that plays crucial roles in physiological and dysfunctional neuronal processes [67]. Irradiation with 810 nm-0.1W can evoke a physiological release of glutamate, which is consistent with our previous work, describing a dose-dependent relationship between delivered energy and released glutamate [52]. This finding suggests that the nerve terminal responds to PBM differently from a biphasic response, as observed in different cell types [68], and appears to be more sensitive to our PBM parameters than endothelial or mesenchymal stromal cells [69,70].

As is well known, glutamate is the primary excitatory neurotransmitter in the brain. Its release occurs through a calcium-dependent exocytosis process that requires energy in the form of ATP. ATP is essential for functioning ion pumps (such as the Na⁺/K⁺ ATPase pump) that maintain the ion gradients required for presynaptic membrane depolarisation and neurotransmitter release. Our previous work highlighted how PBM with 810 nm-1 W and 810 nm-0.1 W acted on the glutamate release process through voltage-operated Ca^2+^ and Na⁺ channel-related mechanisms [51,52].

In this work, we describe the effect of PBM on mitochondrial activity and the relative impact on neurotransmitter release (Figure 4). Indeed, an increase in oxygen consumption and ATP production in synaptosomes is demonstrated following irradiation with 810 nm-1 W and 810 nm-0.1 W (Figure 2). Again, the effect is dose-dependent and keeps the mitochondrial system coupled (Figure 2). Compared to controls, this finding is supported by the observation of no increase in oxidative stress damage (Figure 3) and maintenance of P/O (Figure 2), which describes the efficiency of oxidative phosphorylation in ATP production in mitochondria. On the other hand, previous analyses of isolated mitochondria had identified Complexes IV and III as the targets of PBM at 810 nm [11], with an efficiency of the energy conversion process that was effective but about 10-fold lower than that of chloroplasts during photosynthesis [56]. In contrast, laser treatment appeared ineffective in other dehydrogenase activities, including those belonging to the Krebs cycle [12]. This allows us to speculate that the increased glutamate release observed after laser treatment could depend on increased ATP availability due to the activation of OxPhos rather than modulation of the Krebs cycle via glutamate availability. On the other hand, the concentrations involved are different: when glutamate acts as a neurotransmitter, it is released from synaptosomes in the picomoles range; conversely, when it displays a metabolic function through the Krebs cycle, its concentration reaches the micromoles range.

Our data on PBM and synaptosomes are consistent with what has been observed in *Paramecium*. This non-nerve cell exhibits excitatory stimulus-response capabilities akin to those of more evolved organisms [71]. Like neurons during the transmission of nerve signals, *Paramecium* responds to chemical and physical stimuli through membrane depolarization [72]. PBM with 810 nm induces increased mitochondrial respiration [10], ATP production [73], and membrane depolarization phenomena in *Paramecium* due to its effects on calcium homeostasis [74]. This response to PBM seems to operate according to mechanisms conserved during evolution, uniting even phylogenetically distant excitatory cells. In response to PBM, synaptosomes released glutamate, and the overflow observed was not different from that measured with a depolarizing stimulation such as 4-AP (Figure 4). The not-additive effect of the PBM and 4-AP could be due to the convergence of events at the membrane level: 4-AP is a K^+^-channel blocker, and it reduces the probability of a membrane re-polarization. On the other hand, PBM promotes glutamate release, which is partially inhibited by blocking voltage-operated Ca^2+^ channels or TTX-sensitive Na^+^ channels [52], both of which are involved in neurotransmitter release in response to depolarization. The close correlation between the transformation of photon energy into chemical energy (ATP) and the glutamate release is further supported by our results obtained in the presence of rotenone. In the basal condition, treatment of synaptosomes with rotenone inhibited glutamate release evoked by PBM (Figure 4).

Rotenone and antimycin are inhibitors of mitochondrial function that act on different complexes in the electron transport chain. Rotenone acts on Complex I, preventing electrons from being transferred from NADH to the electron transport chain. However, Complex II (succinate dehydrogenase) can still contribute to electron flow in the chain by transferring electrons from FADH2 to ubiquinone (CoQ) [75]. On the other hand, antimycin acts on Complex III (cytochrome bc1 complex) of the mitochondrial electron transport chain, inhibiting electron transfer from ubiquinol (reduced coenzyme Q) to cytochrome c [76]. This interruption blocks the electron transport chain and stops the oxidative phosphorylation process. Rotenone’s mode of action explains why irradiating synaptosomes treated with rotenone can still stimulate ATP production, releasing glutamate.

PBM at 810 nm re-modulates excitotoxic glutamate release induced by rotenone-dependent mitochondrial dysfunction.

In vitro, cell inhibition with rotenone is used as a model for the study of Parkinson’s disease (PD) as it emulates many features of the neurodegenerative disease [77,78].

We mentioned that Complex I of the electron transport chain in mitochondria is inhibited by rotenone. This effect mimics mitochondrial dysfunction. Indeed, it leads to decreased ATP production, creating energy deficits in neuronal cells like in PD [79]. In addition, inhibition of Complex I can lead to increased production of reactive oxygen species (ROS) due to increased CoQ reduction, which can facilitate electron transfer to oxygen [80,81]. Recapitulating, our data demonstrates that during superfusion on purified cortical synaptosomes, the glutamate release evoked by PBM was sensible to the Complex I activity (Figure 4). When a depolarizing stimulus was co-applied, the release of the neurotransmitter was dysregulated, and the synaptosomes responded with a very high overflow of endogenous glutamate. Previously, it was demonstrated that synaptosomes treated with rotenone for 15 min, with a reduction of Complex I activity, mitochondrial membrane potential and plasma membrane potential, were more susceptible to 4-AP depolarisation [82]. In PD, the Complex I activity has been reduced in post-mortem studies performed on homogenates prepared from the *substantia nigra* [83] and frontal cortex [84]. The mitochondrial dysfunction at nerve terminals and the related effect on the intracellular [Ca^2+^] and [Na^+^] has been related to neuronal cell death [40,85,86,87] as observed in PD [40]. Synaptosome’s increased susceptibility to depolarization is consistent with the high overflow measured when nerve terminals were depolarized in the presence of rotenone. The most relevant data is that, in these conditions, 810 nm PBM restores glutamate release to a near-physiological level. Therefore, it is of great interest to study PBM on rotenone-treated synaptosomes. Our observation could sustain the potential use of PBM in clinical trials on PD patients or to treat other acute or neurodegenerative conditions related to glutamate excitotoxicity, such as traumatic brain injury, stroke, anxiety, or depression for which PBM-t is effective in pre-clinical models [88].

Speculating, the significantly reduced effect of rotenone on ATP production could impair calcium homeostasis, which is no longer effectively controlled, leading to a high neurotoxic release of glutamate when the nerve terminals are stimulated [87,88]. Indeed, in the presence of rotenone, the Ca^2+^-independent release of glutamate from synaptosomes depolarized with 4-AP was increased [89]. In this condition, neurotransmitter release is deregulated, leading to excitotoxicity and promoting the pathogenesis of neurodegenerative disorders. Finally, it could be speculated that the PBM-over production of ATP may bring glutamate release back into the context of a vesicular and calcium-dependent release. This aspect deserves further investigation, accompanied by observations on the efficiency of machinery and on the molecular players involved in exocytosis. Regardless of the pathway taken, 810 nm PBM stimulates ATP production, helping to ease the energetic dysfunction by partly enabling a physiological restoration of neurotransmission functioning.

## 5. Conclusions

In conclusion, we can consider some of our work’s limitations and prospects. The main limitation of using synaptosomes is that they partially represent the in vivo conditions and complexity of the nervous system. However, this isolation has numerous advantages for the detailed and specific analysis of synaptic functions and their effects due to their isolated nature, ease of manipulation, and rapid responses [90,91]. Moreover, in PD, neuronal cell death seems to initiate in the nerve endings [92]. Thus, the chosen biological model may better fit the investigation of the PBM potential effects in the early stages of PD.

This allows us to represent the light-glutamate relationship more extensively by considering evidence reported in the literature. From our data, it is possible to identify the mechanism of action of the 810 nm PBM-t that, through a primary effect on the activity of the mitochondrion in the synaptosome, supports the secondary effect observed as glutamate release. This effect depends on the photo-energy and seems effective both under physiological conditions and under conditions of mitochondrial dysfunction, provided that the Complex II pathway, and thus the production of ATP, is still guaranteed, albeit at a reduced level. On the other hand, authors have described how stimulation of neurons with glutamate can induce the release of photoenergy in the form of biophotons [93,94] in the visible to near-infrared wavelength range up to 800 nm [95]. It has been proposed that the photoenergy emitted by neurons may influence neighbouring cells, potentially activating repair pathways such as the production of growth factors or other molecules that promote cell survival and repair [96,97,98]. Again, mitochondrial activity would play a significant role in cell-biophoton-cell interaction. The totality of the data suggests a ″light code″ that can play a role in PBM-t and photo-neurotransmission, where neurons respond to light and ″communicate″, with mitochondria playing a central role in these processes.

## Data Availability

The data are available on request from the authors.
